# Occurrence and Characterization of NDM-1-Producing *Shewanella* spp. and *Acinetobacter portensis* Co-Harboring *tet*(X3) in a Chinese Dairy Farm

**DOI:** 10.3390/antibiotics11101422

**Published:** 2022-10-17

**Authors:** Ruichao Li, Lifei Zhang, Xiaoyu Lu, Kai Peng, Yuan Liu, Xia Xiao, Hongqin Song, Zhiqiang Wang

**Affiliations:** 1 Jiangsu Co-Innovation Center for Prevention and Control of Important Animal Infectious Diseases and Zoonoses, College of Veterinary Medicine, Yangzhou University, Yangzhou 225009, China; 2 Institute of Comparative Medicine, Yangzhou University, Yangzhou 225009, China; 3 Joint International Research Laboratory of Agriculture and Agri-Product Safety, the Ministry of Education of China, Yangzhou University, Yangzhou 225009, China

**Keywords:** *Acinetobacter portensis*, *Shewanella* spp., *tet*(X3), *bla*_NDM-1_, co-existence

## Abstract

Bacteria with carbapenem or tigecycline resistance have been spreading widely among humans, animals and the environment globally, being great threats to public health. However, bacteria co-carrying drug resistance genes of carbapenem and tigecycline in *Shewanella* and *Acinetobacter* species remain to be investigated. Here, we detected nine *bla*_NDM-1_-carrying *Shewanella* spp. isolates as well as three *A. portensis* isolates co-harboring *tet*(X3) and *bla*_NDM-1_ from seventy-two samples collected from a dairy farm in China. To explore their genomic characteristic and transmission mechanism, we utilized various methods, including PCR, antimicrobial susceptibility testing, conjugation experiment, whole-genome sequencing, circular intermediate identification and bioinformatics analysis. Clonal dissemination was found among three *A. portensis*, of which *tet*(X3) and *bla*_NDM-1_ were located on a novel non-conjugative plasmid pJNE5-X3_NDM-1 (333,311 bp), and the circular intermediate ΔIS*CR2*-*tet*(X3)-*bla*_NDM-1_ was identified. Moreover, there was another copy of *tet*(X3) on the chromosome of *A. portensis*. It was verified that *bla*_NDM-1_ could be transferred to *Escherichia coli* C600 from *Shewanella* spp. by conjugation, and self-transmissible IncA/C_2_ plasmids mediated the transmission of *bla*_NDM-1_ in *Shewanella* spp. strains. Stringent surveillance was warranted to curb the transmission of such vital resistance genes.

## 1. Introduction

Carbapenems are essential treatment options for clinically significant, multidrug-resistant (MDR) Gram-negative bacteria infections because they have a broad antibacterial spectrum and high antibacterial activity [1]. On the other hand, the populations of carbapenem-resistant bacteria have been quickly growing worldwide in recent years, posing a severe threat to public health [2]. Diverse carbapenemases, which are typically encoded on transmissible plasmids, are the fundamental mechanism of carbapenem resistance. The New Delhi metallo-β-lactamase (NDM), *Klebsiella pneumoniae* carbapenemase (KPC) and OXA-48-type oxacillinase are the three most common carbapenemases [3]. The NDM-1 was initially discovered in India and has now spread throughout the world. It has the ability to hydrolyze practically all β-lactam antibiotics, resulting in the development of MDR bacteria [4]. Because of their excellent therapeutic action on extended-spectrum β-lactamases (ESBLs) and the AmpC enzyme-producing bacteria, meropenem and imipenem are frequently used to treat severe Gram-negative bacteria infections. However, due to the wide prevalence of carbapenem-resistant Gram-negative bacteria in recent years, effective antibiotics against drug-resistant bacteria remain scarce, with tigecycline serving as the last-resort option [5].

Tigecycline is used to treat a variety of clinical infections caused by Gram-positive and Gram-negative bacteria with multidrug resistance. However, the discoveries of plasmid-mediated *tet*(X3) and *tet*(X4) have limited its utility, owing to the capacity of *tet*(X) to catalyze the degradation of tigecycline [6,7]. According to previous retrospective screening, plasmid-borne *tet*(X) genes were present in bacteria of different settings, including food animals, migratory birds, clinical specimens and environmental samples in China [7,8]. Importantly, *tet*(X3) also had a high carriage rate in the gastrointestinal tract of cows [9]. Considering that there are many cow-related food products, antibiotic resistance genes could have been transmitted to humans through vocational contact and transfer among humans.

Known as environmental bacteria, *Shewanella* spp. was widely distributed in marine ecosystems and could also be recovered from food-producing animals and human active areas such as hospitals [10,11,12]. Although most of the human infections reported linked with *Shewanella* spp. were opportunistic and sporadic, disease syndromes and multidrug resistance have still increased in recent years [13]. Previous reports about *Shewanella* related to multidrug resistance were usually associated with *bla*_OXA_ rather than *bla*_NDM_. A *bla*_OXA-416_-carrying extensively resistant isolate of *Shewanella* *xiamenensis* was isolated in Algeria from hospital effluents [14], *bla*_OXA-55_-carrying *Shewanella algae* was isolated from a patient in the hospital in Marseille, France [15] and several chromosome-based *bla*_OXA-48_-like variants were found in *Shewanella* spp. from ornamental fish in the Netherlands [10]. In 2017, *Shewanella putrefaciens* with chromosomal *bla*_OXA-436_ and plasmid-borne *bla*_NDM-1_ was isolated from a hospital in Pakistan [11].

*Acinetobacter portensis* was a novel *Acinetobacter* species originally identified from raw meat [16]. It is now further described as one MDR pathogen in this study for the first time. The emergence of *Acinetobacter* spp. co-harboring *tet*(X3) and *bla*_NDM-1_ from animal samples in China has already been reported, but *A. portensis* was not investigated [5,17].

In this study, we looked at the prevalence and molecular characteristics of MDR bacteria with resistance to carbapenem and tigecycline from a dairy farm in China in 2021, and we totally identified twelve NDM-1-producing isolates, including nine *Shewanella* spp. strains and three *A. portensis* strains carrying two copies of *tet*(X) simultaneously, all of which were collected from milking environment samples. The genomic epidemiology of drug-resistant strains and the characteristics of resistance plasmids were also analyzed, with the goal of learning about the molecular genetic characteristics of carbapenem-resistant and tigecycline-resistant bacteria of dairy farm origin to infer the underlying potential public health concerns.

## 2. Results and Discussion

### 2.1. Collection of Antimicrobial-Resistant Strains and Resistance Phenotypes

Thirteen meropenem-resistant strains were isolated from seventy-two non-duplicated samples collected from a dairy farm in China, including three *A. portensis* (23.08%), nine *Shewanella* spp. (69.23%) and one *Stenotrophomonas maltophilia* (7.69%). Except for the strain *Stenotrophomonas maltophilia* with inherent resistance to carbapenems collected from the shed environment, the remaining isolates were all identified from milking environment samples and demonstrated to be *bla*_NDM_-positive by PCR, with three *A. portensis* strains positive for *tet*(X) as well.

The results of antimicrobial susceptibility testing show that all the carbapenem-resistant isolates conferred resistance to meropenem, ceftiofur and amoxicillin but were susceptible to enrofloxacin, tigecycline and colistin. Five isolates showed resistance to chloramphenicol (38.46%) and seven isolates showed resistance to tetracycline (53.85%). Due to inherent drug resistance in *Stenotrophomonas maltophilia*, we only incorporated this strain into antimicrobial susceptibility testing, but no more in-depth explorations were conducted.

Three *A. portensis* isolates exhibited highly similar antimicrobial susceptibility profiles, being resistant to meropenem, ceftiofur, tetracycline and amoxicillin but susceptible to other antibiotics tested (Table 1). *Acinetobacter* spp. carrying *tet*(X) but susceptible to tigecycline was reported previously [9,18]. This phenomenon confirmed it was always possible to detect *tet*(X) in strains without a tigecycline resistance phenotype, which highlighted that the traditional methods of bacterial isolation based on selective media supplemented with antibiotics may impair the recovery of bacteria harboring critical precursors of resistance genes, although they may not confer the resistance phenotype directly. For some reasons to be investigated, one possibility was the inhibition of *tet*(X) function within certain species, suggesting *tet*(X) still could express its resistance by transmitting to other hosts [9,18]. In a manner of speaking, the existence of this silent dissemination was even more dangerous.

All of the *Shewanella* spp. isolates were sensitive to colistin, tigecycline and enrofloxacin but resistant to meropenem, ceftiofur and amoxicillin. Five strains (55.6%) and four strains (44.4%) were resistant to chloramphenicol and tetracycline, respectively. The minimum inhibitory concentrations of *Shewanella* spp. isolates to meropenem were in the range from 16 to 128 μg/mL (Table 1). As previously reported, in *Shewanella*, *bla*_OXA_ could lead to a low-level resistance (8–16 μg/mL) to meropenem or was incapable of reducing susceptibility to carbapenems in different occasions [19,20,21]. By contrast, the production of NDM was more concerned in terms of meropenem resistance in these bacteria.

### 2.2. Transfer Ability of MDR Plasmids

Conjugation experiments by broth mating were conducted on twelve *bla*_NDM_-positive strains. The results show that the *bla*_NDM-1_ of seven *Shewanella* strains (77.8%) could be transferred to the recipient strain *E. coli* C600. Antimicrobial susceptibility testing demonstrated that the corresponding transconjugants were resistant to amoxicillin, meropenem and ceftiofur, and MICs of meropenem ranged from 16 to 32 μg/mL (Table 1). The fact that the carbapenem resistance gene *bla*_NDM-1_ could spread among different species of bacteria suggested *Shewanella* spp. was likely to be the reservoir of antibiotic resistance genes, including *bla*_NDM-1_.

For three *A. portensis*, both *tet*(X3) and *bla*_NDM-1_ genes could not be transferred to three different recipient strains by broth mating. Subsequently, we performed electroporation experiments on these strains but also failed after three repeats. Recently, isolates bearing *tet*(X) without corresponding tigecycline resistance have been reported [9,18]. Plasmidscarrying *tet*(X3) could be transferred to *Acinetobacter baumannii* from *Acinetobacter towneri* susceptible to tigecycline, making a 128-fold increase in the MIC of tigecycline in transconjugant. This species-dependent peculiarity was likely to explain the different resistance phenotypes conferred by the same gene [18]. However, plasmids carrying *tet*(X3) and *bla*_NDM-1_ in the same *Acinetobacter* species were often non-conjugative in lab experiment conditions [9].

### 2.3. Characteristics of Nine Shewanella spp. Isolates

**Figure 1:**
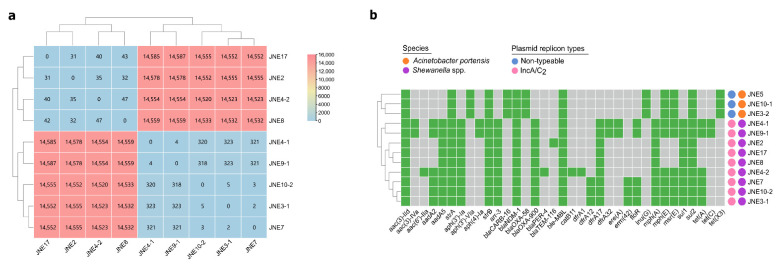
Characteristics of thirteen carbapenem-resistant isolates. (**a**) A heatmap of core genome SNPs analysis among nine *Shewanella* spp. strains carrying *bla*_NDM-1_. (**b**) A heatmap of antibiotic resistance genes, species and plasmid replicon types for thirteen carbapenem-resistant isolates. Resistance genes are marked positive by green and negative by gray. The species and plasmid replicon types are showed by different colored circles.

For better characterization, the genomic DNA of nine *Shewanella* spp. isolates were extracted then subjected to short-read and long-read sequencing. Species identification by whole-genome sequences based on PubMLST displayed that the nine *Shewanella* strains had the highest match with *Shewanella putrefaciens* (52~60%). Considering the matching degrees were not high enough, we thought conservatively that these nine *Shewanella* strains belonged to other subspecies rather than *Shewanella putrefaciens*. There were only small SNPs among the core genomes of JNE2, JNE17, JNE4-2 and JNE8 (Figure 1a) and highly similar antibiotic resistance gene distribution (Figure 1b), indicating these four strains from different samples had a close phylogenetic relationship and may derive from the same ancestor. Likewise, there were no obvious SNPs differences among JNE4-1, JNE9-1, JNE10-2, JNE3-1 and JNE7 (Figure 1a), which means the five strains were clonally related. However, significant differences lay between these two groups of strains (SNPs > 14,500) (Figure 1a). Not only did this suggest that these two groups of strains are clearly not from the same clone, but their subspecies were also likely to be different. Based on this, we tended to classify these nine strains into two new distinct subspecies of *Shewanella*.

There were at least 14 resistance genes in every *Shewanella* strain, and a multicopy of *sul1* existed in these strains (Figure 1b). This further implied *Shewanella* spp. may well be the reservoir of significant antibiotic resistance genes, facilitating the wide spread of drug-resistant bacteria. Replicon analysis found that all of the *Shewanella* isolates carried plasmids of IncA/C_2_ type (Figure 1b). Over the years, the spread of *bla*_NDM_ has been found associated with IncA/C plasmids [22,23].

**Figure 2:**
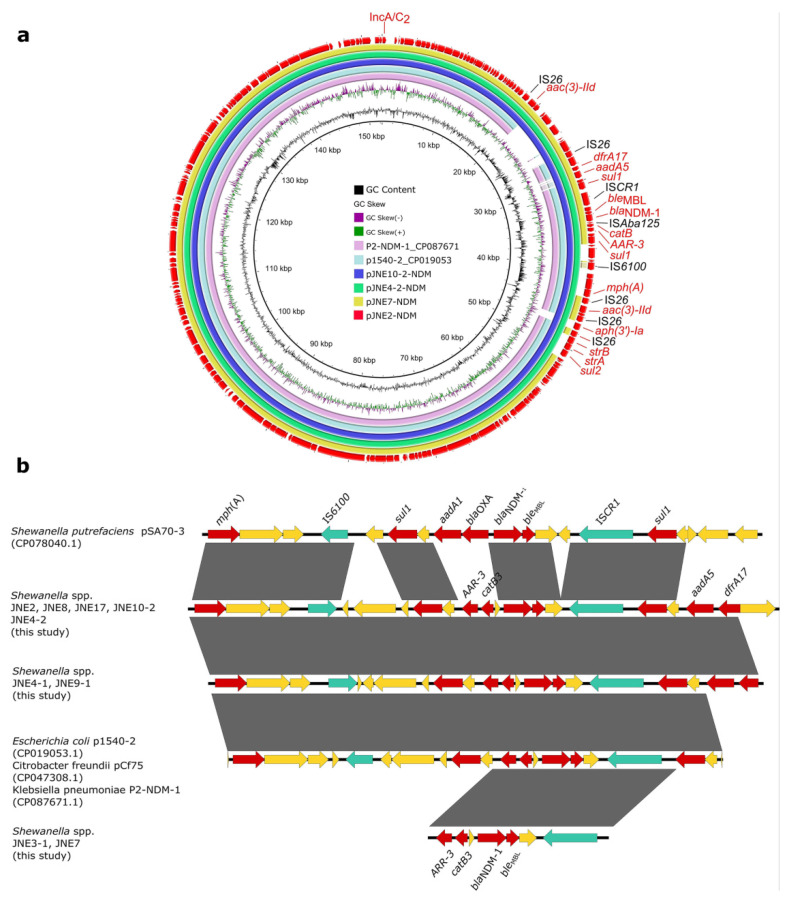
Genetic features of *bla*_NDM-1_ in *Shewanella* strains. (**a**) Circular comparison of *bla*_NDM-1_-carrying plasmids pJNE2-NDM, pJNE7-NDM, pJNE4-2-NDM and pJNE10-2-NDM. (**b**) Linear sequence comparison of genetic context of *bla*_NDM-1_ in the nine *Shewanella* spp. isolates in this study with pSA70-3 (CP078040.1), p1540-2 (CP019053.1), pCf75 (CP047308.1) and P2-NDM-1 (CP087671.1) from the NCBI database. Regions with >99% sequence identity are marked by gray shading, and red arrows denote antibiotic resistance genes. Green arrows denote insertion sequences, and yellow arrows represent other genes.

In line with SNPs analysis, JNE7, JNE2, JNE4-2 and JNE10-2 were chosen as the representative strains to be sequenced with the MinION long-read platform. Results show that *bla*_NDM-1_ in four strains was located on IncA/C_2_ plasmids, pJNE7-NDM (137,224 bp), pJNE2-NDM (152,348 bp), pJNE4-2-NDM (152,348 bp) and pJNE10-2-NDM (166,090 bp), respectively. Based on the WGS analysis, it was found that clonal dissemination existed among 9 *Shewanella* isolates, and it could be concluded that *bla*_NDM-1_ located on IncA/C_2_ plasmids was prevalent in nine *Shewanella* isolates. The four plasmids showed high similarity, and pJNE2-NDM showed 100% sequence identity to the plasmid p1540-2 (94% coverage, CP019053) in *E. coli* and 99.98% sequence identity to the plasmid P2-NDM-1 (94% coverage, CP087671) in *K. pneumoniae* (Figure 2a), suggesting the dissemination of *bla*_NDM-1_ in milking environments was mediated by highly similar IncA/C_2_ plasmids and highlighting the mobile nature of those resistance genes. We subsequently analyzed the detailed genetic contexts of *bla*_NDM-1_ by mapping the assembled sequences to the plasmid pSA70-3 in *Shewanella putrefaciens*, p1540-2 in *E. coli*, pCf75 in *Citrobacter freundii* and P2-NDM-1 in *K. pneumoniae* from the NCBI database (Figure 2b). Limited by the short-read data, complete accurate genetic structures were difficult to obtain. However, it could still be observed clearly that the genetic contexts of *bla*_NDM-1_ among strains in this study and those from the database were strikingly similar, revealing the wide spread of *bla*_NDM-1_ among different bacteria.

### 2.4. Characteristics of Three Acinetobacter Portensis Isolates

**Figure 3:**
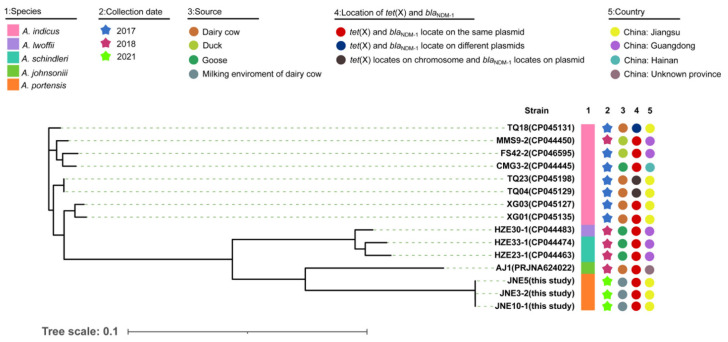
Phylogenetic analysis and genetic characteristics of *Acinetobacter* isolates co-harboring *tet*(X) and *bla*_NDM-1_ from this study and the NCBI database. The strain species, collection date, source, country and location of *tet*(X) and *bla*_NDM-1_ in the genomes are showed by subsequent color blocks.

Three *A. portensis* strains co-carrying *tet*(X3) and *bla*_NDM-1_ were isolated from different milking environment samples, which were designated as JNE5, JNE3-2 and JNE10-1, respectively. The phylogenetic analysis based on SNPs of core genomes showed that three *A. portensis* strains had a high degree of similarity (Figure 3), and there were no more than 22 SNPs among core genomes of these strains, implying clonal dissemination was likely to occur in the dairy farm.

For better comparison, we gathered information of *Acinetobacter* isolates co-harboring *tet*(X3) and *bla*_NDM-1_ in recent years from an online database (Figure 3). According to the information collected, including the data of this study, *Acinetobacter indicus* carrying both *tet*(X3) and *bla*_NDM-1_ had been isolated as early as 2017 in China. However, the locations of *tet*(X3) and *bla*_NDM-1_ were not conserved, either on the same plasmid, on different plasmids, or on chromosome and plasmid separately (Figure 3). Such co-existing isolates were concentrated in China and sporadically distributed; they were diverse in species, with the majority of them belonging to *A. indicus* (Figure 3). Additionally, these isolates came from a wide range of sources, including dairy cows and their environments, ducks and gooses. *A. portensis* was a newly isolated species of *Acinetobacter* that harbored both *tet*(X3) and *bla*_NDM-1_; this was the first time that such drug-resistant bacteria had been collected from the milking environment of dairy cows. In the *A. portensis* in this study, *tet*(X3) and *bla*_NDM-1_ were located on the same plasmid, similar to the majority of such co-existing stains collected in recent years (Figure 3). Considering the diverse sources and wide distribution of strains in China carrying both *tet*(X3) and *bla*_NDM-1_, and the situation that there was probably clonal dissemination in this study, measures must be implemented to avoid their further dissemination and contamination.

Interestingly, each *A. portensis* isolate carried 13 identically acquired antibiotic resistance genes (Figure 1b), conferring resistance to aminoglycosides (*aac(3)-IId*, *aph(3′)-Via*, *strA*, *strB*), sulphonamide (*sul2*), glycopeptides (*ble*_MBL_), macrolide (*mph*(E)*, msr*(E)), lincosamides (*lnu*(G)), carbapenems (*bla*_NDM-1_, *bla*_CARB-16_ and *bla*_OXA-58_) and tetracyclines (*tet*(X3)), further supporting the aforementioned clonal dissemination.

### 2.5. Genetic Contexts of bla_NDM-1_ and tet(X3)

**Figure 4:**
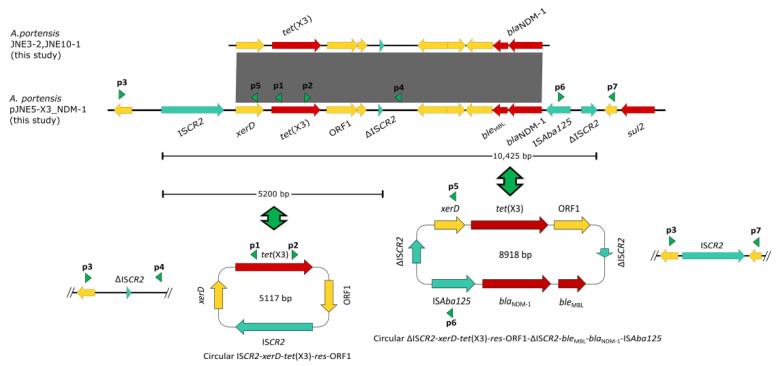
Linear sequence comparison of genetic context of *tet*(X3) and *bla*_NDM-1_ in three *Acinetobacter portensis* isolates. Regions of >99% homology are marked by gray shading, red arrows denote antibiotic resistance genes and arrows indicate the direction of transcription of the genes. Green arrowheads p1, p2, p3 and p4 indicate the positions of primers used for amplification of the IS*CR2*-*xerD*-*tet*(X3)-*res*-ORF1 circular intermediate, arrowheads p3, p5, p6 and p7 are used for amplification of the ΔIS*CR2*-*xerD*-*tet*(X3)-*res*-ORF1-ΔIS*CR2*-*ble*_MBL_-*bla*_NDM-1_-IS*Aba125* circular intermediate.

In the same way, we performed short-read sequencing of three *A. portensis*. According to the linear comparison of sequences, it was obvious that the genetic contexts of *bla*_NDM-1_ and *tet*(X3) in JNE5, JNE3-2 and JNE10-1 were identical (Figure 4). On account of the clonal dissemination among three *A. portensis* strains revealed by phylogenetic analysis, we chose the JNE5 strain as the representative strain to be sequenced with the MinION long-read platform to obtain the complete circular genome sequences. Analysis displayed that the genome of JNE5 was comprised of a chromosome (2,568,515 bp) and five non-typeable plasmids, pJNE5-X3_NDM-1 (333,311 bp), pJNE5-64k (64,629 bp), pJNE5-OXA-58 (59,583 bp), pJNE5-3k (3617 bp) and pJNE5-1k (1416 bp). Most of the drug resistance genes were located on plasmid pJNE5-X3_NDM-1. In addition, it should be noted that *tet*(X3) had two copies, one was located on the chromosome, and the other one was located on the plasmid pJNE5-X3_NDM-1 with *bla*_NDM-1_. Moreover, there was one *bla*_OXA-58_ existing in pJNE5-OXA-58.

**Figure 5:**
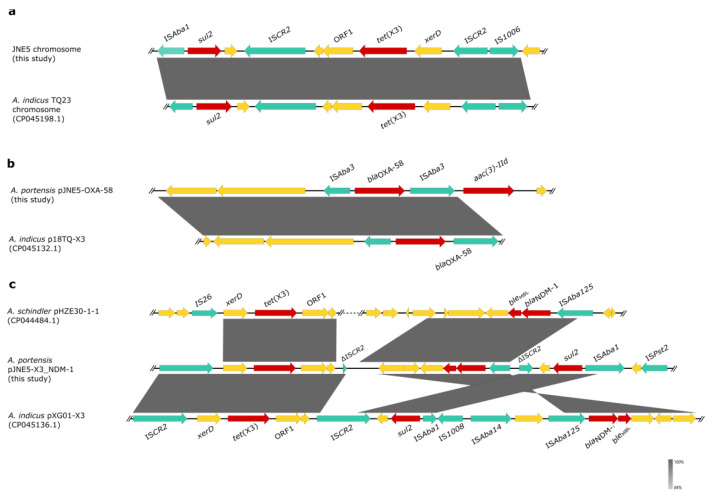
Genetic features of *bla*_NDM-1_, *tet*(X) and *bla*_OXA-48_ in JNE5 strain. (**a**) Linear alignment of the *tet*(X3)-carrying chromosome in JNE5 with chromosome from *A. indicus* TQ23 (CP045198.1). (**b**) Linear alignment of the *bla*_OXA-48_-positive plasmid pJNE5-OXA-58 with p18TQ-X3. (**c**), Linear sequence comparison of genetic context of *bla*_NDM-1_ and *tet*(X3) in plasmid pJNE5-X3_NDM-1 with that of pHZE30-1 (CP044484.1) and pXG01-X3 (CP045136.1). Regions of homology are marked by shading; red arrows denote antibiotic resistance genes. Green arrows denote insertion sequences, and yellow arrows represent other genes.

**Figure 6:**
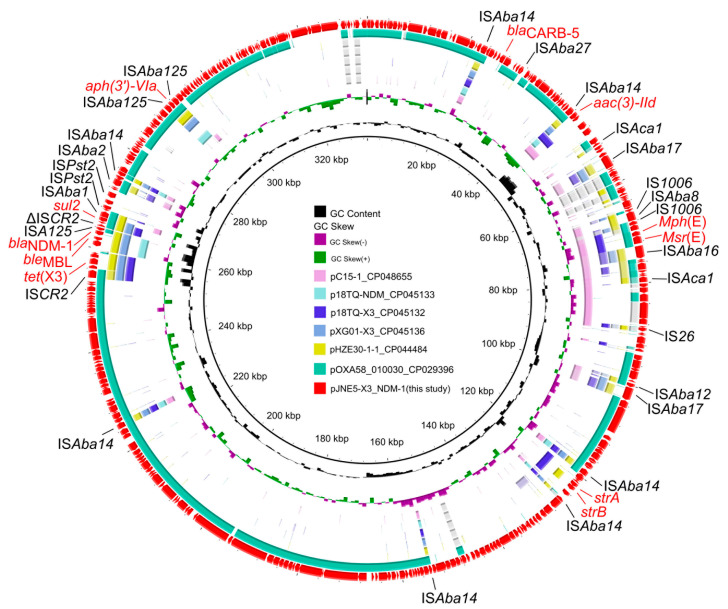
Circular comparison of the plasmid pJNE5-X3_NDM-1 co-harbouring *tet*(X3) and *bla*_NDM-1_ in *Acinetobacter portensis* JNE5 strain with similar plasmids from the NCBI database. The outmost circle denotes the reference plasmid pJNE5-X3_NDM-1.

The genetic context of chromosomal *tet*(X) had high coverage with that of the *A. indicus* TQ23 chromosome (CP045198.1). Similarly, genetic contexts of *bla*_OXA-58_ on plasmid pJNE5-OXA-58 and *A. indicus* p18TQ-X3 (CP045132.1) separately also had a large number of consistencies (Figure 5a,b). Plasmid comparison revealed that the plasmid pJNE5-X3_NDM-1 shared a low degree of genetic identity with two previously reported plasmids co-harboring *tet*(X3) and *bla*_NDM-1_ (pXG01-X3 and pHZE30-1-1). Although pOXA58_010030 showed the highest sequence identity to pJNE5-X3_NDM-1, it lacked the most important multidrug resistance region (Figure 6). The typical genetic context, IS*Aba125*-*bla*_NDM-1_-*ble*_MBL_-*trpF*-*dsbC*, appeared in plasmid pJNE5-X3_NDM-1 [24]. Additionally, *tet*(X3) was located within a 5200 bp region with the gene arrangement IS*CR2*-*xerD*-*tet*(X3)-*res*-ORF1-ΔIS*CR2*, with one intact IS*CR2* element located on the upstream of *tet*(X3) and another ΔIS*CR2* (83 bp) with the same orientation lay in the downstream (Figure 4).

Inverse PCR was carried out to examine whether the minicircle IS*CR2*-*tet*(X3) could take shape. Finally, we obtained a fragment of 4245 bp in length, and sequence analysis revealed that it included only one intact copy of IS*CR2* element (IS*CR2*-*xerD*-*tet*(X3)-*res*-*orf1*) (Figure 4), which was the same as previously reported [9]. To reconfirm the above situation, we used inward-facing primers and then obtained a 1664 bp PCR amplicon, which included only one ΔIS*CR2* (83 bp) element (Figure 4). Compared with other reported plasmids co-harboring *tet*(X3) and *bla*_NDM-1_, the significant difference in plasmid pJNE5-X3_NDM-1 was that *tet*(X3) and *bla*_NDM-1_ stood exceptionally close to each other (Figure 5c). What was noteworthy was that one ΔIS*CR2* (389 bp) element lay in the downstream of *bla*_NDM-1_; since it was in the same direction as the intact IS*CR2* element located on the upstream of *tet*(X3), outward-facing primers were designed to examine whether this MDR region could also be excised and mobilizable. A 1875 bp amplicon was generated and sequence analysis revealed that it included only one copy of the ΔIS*CR2* (389 bp) element. The results of PCR using inward-facing primers showed a 2933 bp amplicon, including one intact copy of the IS*CR2* element, which verified the instability of circular intermediate ΔIS*CR2*-*tet*(X3)-*bla*_NDM-1_ by excision (Figure 4). The generation of circular intermediate containing *tet*(X3) and *bla*_NDM-1_ was a warning that the two important antibiotic resistance genes were likely to transmit together, which warranted further investigations. Although conjugation experiments failed, the potential hazard of the MDR plasmids still could not be neglected.

## 3. Materials and Methods

### 3.1. Bacterial Isolation and Identification

In total, 72 non-duplicated samples were collected from a dairy farm in Xuzhou, Jiangsu Province in May 2021, including 14 milk samples from cows with mastitis, 31 stool samples, 15 milking environment samples and 12 shed environment samples. To enrich the microbiota, solid and liquid samples, as well as cotton swabs (surface samples), were incubated in a 5 mL LB broth without antimicrobials for 6 h. These enrichment broth suspensions were streaked onto MacConkey agar plates containing meropenem (2 mg/L) using a sterile loop to isolate carbapenem-resistant colonies. The boiling method was used to extract bacterial genome DNA, which was subsequently screened for carbapenem resistance genes (*bla*_NDM_, *bla*_VIM_, *bla*_KPC_, *bla*_IMP_, *bla*_SIM_, *bla*_DIM_, *bla*_AIM_, *bla*_BIC_, *bla*_SPM_, *bla*_OXA_ and *bla*_GIM_) using the previously reported primers [25]. The carbapenem-resistant bacteria were screened again for *tet*(X) genes using primers described earlier [6]. 16S rRNA gene sequencing was conducted to further identify their species using the forward primer 5′-AGAGTTTGATCATGGCTCAG-3′ and the reverse primer 5′-GTGTGACGGGCGGTGTGTAC-3′.

### 3.2. Antimicrobial Susceptibility Testing

Fresh CAMH broth was prepared for antimicrobial susceptibility testing, and the minimal inhibitory concentrations (MICs) of chloramphenicol, colistin, meropenem, tigecycline, enrofloxacin, ceftiofur, tetracycline and amoxicillin were determined by the broth microdilution method. The results are interpreted according to the guidelines of the Clinical and Laboratory Standards Institute [26] and the European Committee on Antimicrobial Susceptibility Testing (EUCAST, v12.0) (http://www.eucast.org/clinical_breakpoints/ (accessed on 1 January 2022)) with *E. coli* ATCC 25922 as a quality control strain.

### 3.3. Conjugation and Electroporation Experiments

Conjugation experiments by broth mating were carried out using *bla*_NDM_-positive strains as donors, with strains *E. coli* C600 (rifampicin resistant), *E. coli* J53 (sodium azide resistant) and *A. baumannii* ATCC19606 (chloramphenicol resistant) as recipients. Only *E. coli* C600 was used as the recipient of nine *Shewanella* spp. isolates, while all recipients mentioned above were used for *A. portensis*. Transconjugants were separately selected on LB agar plates containing rifampicin (300 mg/L) with meropenem (2 mg/L), sodium azide (300 mg/L) with meropenem (2 mg/L), or chloramphenicol (64 mg/L) together with meropenem (2 mg/L) and then confirmed by PCR. The broth microdilution method was used to determine the MICs of a range of antimicrobials for the transconjugants. For *A. portensis* isolates that meropenem resistance phenotype failed to transfer by conjugation, electroporation experiment was performed, in which *A. baumannii* ATCC19606 was prepared as the receipt strain.

### 3.4. Whole-Genome Sequencing and Bioinformatics Analysis

Genomic DNA of all *bla*_NDM_-positive isolates from overnight cultures was extracted using the FastPure^®^ Bacteria DNA Isolation Mini Kit (Vazyme, Nanjing, China). The genomic DNA was subjected to short-read sequencing (2 × 150 bp) with the Illumina HiSeq 2500 platform (Illumina, San Diego, CA, USA). Short-read Illumina raw reads were de novo assembled using SPAdes [27] with default parameters, and contigs less than 200 bp were discarded. To obtain the complete sequences, we selected several representative strains and extracted their genomic DNA to perform Oxford Nanopore Technologies MinION long-read sequencing [28,29]. The complete genome sequences were modified manually and automatically annotated using RAST (http://rast.nmpdr.org/ (accessed on 1 January 2022)). The BRIG tool was used to perform circular comparison of the plasmids in this study and the homologous plasmids available from the NCBI database [30]. A pairwise SNP distance matrix was generated using snp-dists 0.6.3 (https://github.com/tseemann/snp-dists (accessed on 1 January 2022)). Roary [31] and FastTree [32] based on the SNPs of core genomes were used to construct phylogenetic trees, which were visualized by iTOL v5 [33]. Easyfig was used to generate linear comparison in order to visualize the sequence comparison features of genetic contexts [34].

### 3.5. Identification of Circular Intermediates

To determine whether the recombination of IS*CR2* and ΔIS*CR2* (83 bp) elements could form the *tet*(X3)-carrying circular intermediate, all plasmids, including the *tet*(X3)-carrying plasmid pJNE5-X3_NDM-1 from the JNE5 strain, were extracted for inverse PCR assays, using outward-facing primers p1 5′-TCGGTCGTTGTCTCTTTCGT-3′ and p2 5′-TTGATGTCGCCTTTTGCAGG-3′ for detection of minicircle IS*CR2*-*tet*(X3). When the band of target fragment was detected through agarose gel electrophoresis, the same primers were used to sequence the amplified minicircle product. Then inward-facing primers p3 5′-CGCAGCGTTTCGTACATCAG-3′ and p4 5′-AGGTCAATCAGACTGGGCGTT-3′ were used to verify the excision result. In the same way, to see if the recombination of IS*CR2* and ΔIS*CR2* (389 bp) could lead to the formation of circular intermediate co-carrying *tet*(X3) and *bla*_NDM-1_, outward-facing primers p5 5′-TGTTCCATTCCCTTGGTGGT-3′ and p6 5′-ATGTGCCTTTTTGCCAGGGT-3′ were used for detecting minicircle ΔIS*CR2*-*tet*(X3)-*bla*_NDM-1_. Inward-facing primers p3 and p7 5′-GACGGTATTCGTGGCAAAGC-3′ were used to confirm the excision result.

## 4. Conclusions

In this work, we detected nine NDM-1-producing *Shewanella* spp. strains and three *A. portensis* strains co-harboring *tet*(X3) and *bla*_NDM-1_. Clonal dissemination existed among three *A. portensis* isolates, in which *tet*(X3) and *bla*_NDM-1_ co-located on a novel non-conjugative plasmid, and there was another *tet*(X3) located on the chromosome. Additionally, we confirmed that the circular intermediate ΔIS*CR2*-*tet*(X3)-*bla*_NDM-1_ could be generated. The emergence of *tet*(X3) and *bla*_NDM-1_-bearing plasmids in different bacteria among dairy cow farming environments constitutes a potential public health concern. Continuous monitoring and surveillance of critical resistance genes in such environments are necessary to ensure good farming standards.

## Figures and Tables

**Table 1:** Antimicrobial susceptibility testing of carbapenem-resistant isolates collected from the dairy farm and their corresponding transconjugants.

Strain	Species	MICs (mg/L)							
		CHL	COL	MEM	TGC	ENR	CFF	TET	AMX
C600	*E. coli* (recipient)	4	≤0.25	≤0.25	≤0.25	≤0.25	≤0.25	1	2
JNE5	*Acinetobacter portensis*	1	≤0.25	32	≤0.25	≤0.25	128	16	>128
JNE3-2	*Acinetobacter portensis*	0.5	≤0.25	32	≤0.25	≤0.25	128	16	>128
JNE10-1	*Acinetobacter portensis*	1	≤0.25	32	≤0.25	≤0.25	128	32	>128
JNE10-2	*Shewanella* spp.	32	≤0.25	32	≤0.25	≤0.25	128	32	>128
JNE9-1	*Shewanella* spp.	64	≤0.25	64	0.5	≤0.25	128	32	>128
CJNE9-1	*E. coli* (transconjugant)	8	≤0.25	32	≤0.25	≤0.25	>128	1	>128
JNE8	*Shewanella* spp.	1	≤0.25	64	≤0.25	≤0.25	>128	1	>128
CJNE8	*E. coli* (transconjugant)	8	≤0.25	32	≤0.25	≤0.25	>128	1	>128
JNE3-1	*Shewanella* spp.	64	≤0.25	64	≤0.25	≤0.25	128	16	>128
CJNE3-1	*E. coli* (transconjugant)	4	≤0.25	32	≤0.25	≤0.25	>128	1	>128
JNE17	*Shewanella* spp.	2	≤0.25	128	≤0.25	≤0.25	128	1	>128
CJNE17	*E. coli* (transconjugant)	4	≤0.25	32	≤0.25	≤0.25	>128	2	>128
JNE2	*Shewanella* spp.	2	≤0.25	64	≤0.25	≤0.25	128	0.5	>128
CJNE2	*E. coli* (transconjugant)	4	≤0.25	32	≤0.25	≤0.25	>128	2	>128
JNE7	*Shewanella* spp.	32	≤0.25	16	≤0.25	≤0.25	64	8	>128
CJNE7	*E. coli* (transconjugant)	4	≤0.25	16	≤0.25	≤0.25	>128	1	>128
JNE4-1	*Shewanella* spp.	32	≤0.25	32	0.5	≤0.25	64	16	>128
JNE4-2	*Shewanella* spp.	1	≤0.25	64	0.5	≤0.25	>128	1	>128
CJNE4-2	*E. coli* (transconjugant)	4	≤0.25	32	≤0.25	≤0.25	>128	1	>128
PS ES	*Stenotrophomonas maltophilia*	8	≤0.25	64	0.5	≤0.25	128	8	>128

Abbreviations: CHL, chloramphenicol; COL, colistin; MEM, meropenem; TGC, tigecycline; ENR, enrofloxacin; CFF, ceftiofur; TET, tetracycline; AMX, amoxicillin.

## Data Availability

The draft genome sequences were submitted to NCBI with the BioProject number PRJNA828415. Complete genome sequences of four representative strains JNE5 (PRJNA828443), JNE10-2 (PRJNA829047), JNE2 (PRJNA829244) and JNE7 (PRJNA829126) were also deposited. The complete sequence of pJNE4-2-NDM was also submitted for reference (ON391944).

## References

[1] Papp-Wallace K.M., Endimiani A., Taracila M.A., Bonomo R.A. (2011). Carbapenems: Past, Present, and Future. Antimicrob. Agents Chemother..

[2] Wang H., Li X., Liu B.T. (2021). Occurrence and characterization of KPC-2-producing ST11 Klebsiella pneumoniae isolate and NDM-5-producing Escherichia coli isolate from the same horse of equestrian clubs in China. Transbound. Emerg. Dis..

[3] Kelly A.M., Mathema B., Larson E.L. (2017). Carbapenem-resistant Enterobacteriaceae in the community: A scoping review. Int. J. Antimicrob. Agents.

[4] Wang T., Xu K., Zhao L., Tong R., Xiong L., Shi J. (2021). Recent research and development of NDM-1 inhibitors. Eur. J. Med. Chem..

[5] Cui C.Y., Chen C., Liu B.T., He Q., Wu X.T., Sun R.Y., Zhang Y., Cui Z.H., Guo W.Y., Jia Q.L. (2020). Co-occurrence of Plasmid-Mediated Tigecycline and Carbapenem Resistance in Acinetobacter spp. from Waterfowls and Their Neighboring Environment. Antimicrob. Agents Chemother..

[6] He T., Wang R., Liu D., Walsh T., Zhang R., Lv Y., Ke Y., Ji Q., Wei R., Liu Z. (2019). Emergence of plasmid-mediated high-level tigecycline resistance genes in animals and humans. Nat. Microbiol..

[7] Sun J., Chen C., Cui C.Y., Zhang Y., Liu Y.H. (2019). Plasmid-encoded *tet*(X) genes that confer high-level tigecycline resistance in *Escherichia coli*. Nat. Microbiol..

[8] Cao J., Wang J., Wang Y., Wang L., Gao G.F. (2020). Tigecycline resistance *tet*(X3) gene is going wild. Biosaf. Health.

[9] Zhang R., Dong N., Zeng Y., Shen Z., Lu J., Liu C., Huang Z.A., Sun Q., Cheng Q., Shu L. (2020). Chromosomal and Plasmid-Borne Tigecycline Resistance Genes *tet*(X3) and *tet*(X4) in Dairy Cows on a Chinese Farm. Antimicrob. Agents Chemother..

[10] Ceccarelli D., Essen-Zandbergen A.v., Veldman K.T., Tafro N., Haenen O., Mevius D.J. (2017). Chromosome-Based blaOXA-48-Like Variants in Shewanella Species Isolates from Food-Producing Animals, Fish, and the Aquatic Environment. Antimicrob. Agents Chemother..

[11] Potter R.F., D’Souza A.W., Wallace M.A., Shupe A., Patel S., Gul D., Kwon J.H., Andleeb S., Burnham C.-A.D., Draft G.D. (2017). Genome Sequence of the blaOXA-436 and blaNDM-1-Harboring Shewanella putrefaciens SA70 Isolate. Genome Announc..

[12] Janda J.M., Abbott S.L. (2014). The genus Shewanella: From the briny depths below to human pathogen. Crit. Rev. Microbiol..

[13] Yousfi K., Bekal S., Usongo V., Touati A. (2017). Current trends of human infections and antibiotic resistance of the genus Shewanella. Eur. J. Clin. Microbiol. Infect. Dis..

[14] Yousfi K., Touati A., Lefebvre B., Fournier É., Côté J., Soualhine H., Walker M., Bougdour D., Tremblay C., Bekal S. (2017). A Novel Plasmid, pSx1, Harboring a New Tn1696 Derivative from Extensively Drug-Resistant Shewanella xiamenensis Encoding OXA-416. Microb. Drug Resist..

[15] Cimmino T., Olaitan A., Rolain J. (2016). Whole genome sequence to decipher the resistome of Shewanella algae, a multidrug-resistant bacterium responsible for pneumonia, Marseille, France. Expert Rev. Anti-Infect. Ther..

[16] Carvalheira A., Gonzales-Siles L., Salvà-Serra F., Lindgren S., Moore E.R.B. (2020). *Acinetobacter portensis* sp. nov. and *Acinetobacter guerrae* sp. nov., isolated from raw meat. Int. J. Syst. Evol. Microbiol..

[17] He T., Li R., Wei R., Liu D., Bai L., Zhang L., Gu J., Wang R., Wang Y. (2020). Characterization of Acinetobacter indicus co-harbouring *tet*(X3) and blaNDM-1 of dairy cow origin. J. Antimicrob. Chemother..

[18] Cheng Y.-Y., Liu Y., Chen Y., Huang F.-M., Chen R.-C., Xiao Y.-H., Zhou K., Downing T., Cevallos M., Siddavattam D. (2021). Sporadic Dissemination of *tet*(X3) and *tet*(X6) Mediated by Highly Diverse Plasmidomes among Livestock-Associated Acinetobacter. Microbiol. Spectr..

[19] Tacão M., Araújo S., Vendas M., Alves A., Henriques I. (2018). Shewanella species as the origin of bla genes: Insights into gene diversity, associated phenotypes and possible transfer mechanisms. Int. J. Antimicrob. Agents.

[20] Antonelli A., Di Palo D.M., Galano A., Becciani S., Montagnani C., Pecile P., Galli L., Rossolini G.M. (2015). Intestinal carriage of Shewanella xiamenensis simulating carriage of OXA-48–producing Enterobacteriaceae. Diagn. Microbiol. Infect. Dis..

[21] Tacão M., Correia A., Henriques I. (2013). Environmental Shewanella xiamenensis strains that carry bla OXA-48 or bla OXA-204 genes: Additional proof for bla OXA-48-like gene origin. Antimicrob. Agents Chemother..

[22] Kumarasamy K.K., Toleman M.A., Walsh T.R., Bagaria J., Woodford N. (2010). Emergence of a new antibiotic resistance mechanism in India, Pakistan, and the UK: A molecular, biological, and epidemiological study. Lancet Infect. Dis..

[23] Marta A., Manuel O., Luísa G., Augusto C., Patrice N., Laurent P. (2020). Occurrence of NDM-1-producing Morganella morganii and Proteus mirabilis in a single patient in Portugal: Probable in vivo transfer by conjugation. J. Antimicrob. Chemother..

[24] Fu Y., Du X., Ji J., Chen Y., Jiang Y., Yu Y. (2012). Epidemiological characteristics and genetic structure of blaNDM-1 in non-baumannii Acinetobacter spp. in China. J. Antimicrob. Chemother..

[25] Poirel L., Walsh T.R., Cuvillier V., Nordmann P. (2011). Multiplex PCR for detection of acquired carbapenemase genes. Diagn. Microbiol. Infect. Dis..

[26] CLSI (2020). Performance Standards for Antimicrobial Susceptibility Testing.

[27] Bankevich A., Nurk S., Antipov D., Gurevich A.A., Dvorkin M., Kulikov A.S., Lesin V.M., Nikolenko S.I., Pham S., Prjibelski A.D. (2012). SPAdes: A New Genome Assembly Algorithm and Its Applications to Single-Cell Sequencing. J. Comput. Biol..

[28] Li R., Xie M., Dong N., Lin D., Yang X., Yin W., Wai-Chi C.E., Chen S. (2018). Efficient generation of complete sequences of MDR-encoding plasmids by rapid assembly of MinION barcoding sequencing data. Gigascience.

[29] Wick R.R., Judd L.M., Gorrie C.L., Holt K.E. (2017). Unicycler: Resolving bacterial genome assemblies from short and long sequencing reads. PLoS Comput. Biol..

[30] Alikhan N.F., Petty N.K., Zakour N.L.B., Beatson S.A.A. (2011). BLAST Ring Image Generator (BRIG): Simple prokaryote genome comparisons. Bmc Genom..

[31] Page A.J., Cummins C.A., Hunt M., Wong V.K., Parkhill J. (2015). Roary: Rapid large-scale prokaryote pan genome analysis. Bioinformatics.

[32] Price M., Dehal P., Arkin A. (2009). FastTree: Computing large minimum evolution trees with profiles instead of a distance matrix. Mol. Biol. Evol..

[33] Letunic I., Bork P. (2021). Interactive Tree Of Life (iTOL) v5: An online tool for phylogenetic tree display and annotation. Nucleic Acids Res..

[34] Beatson S.A. (2011). Easyfig: A genome comparison visualizer. Bioinformatics.

